# Transcriptome of Extracellular Vesicles: State-of-the-Art

**DOI:** 10.3389/fimmu.2019.00202

**Published:** 2019-02-28

**Authors:** Andrey Turchinovich, Oxana Drapkina, Alexander Tonevitsky

**Affiliations:** ^1^SciBerg e.Kfm, Mannheim, Germany; ^2^Molecular Epidemiology C080, German Cancer Research Center, Heidelberg, Germany; ^3^National Medical Research Center for Preventive Medicine, Moscow, Russia; ^4^Department of Cell Biology, Higher School of Economics, Moscow, Russia; ^5^Shemyakin-Ovchinnikov Institute of Bioorganic Chemistry RAS, Moscow, Russia; ^6^SRC BioClinicum, Moscow, Russia

**Keywords:** apoptotic bodies, microvesicles, circulating RNA, next generation sequencing, exosomes, extracellular vesicle (EV)

## Abstract

Exosomes and microvesicles are two major categories of extracellular vesicles (EVs) released by almost all cell types and are highly abundant in biological fluids. Both the molecular composition of EVs and their release are thought to be strictly regulated by external stimuli. Multiple studies have consistently demonstrated that EVs transfer proteins, lipids and RNA between various cell types, thus mediating intercellular communication, and signaling. Importantly, small non-coding RNAs within EVs are thought to be major contributors to the molecular events occurring in the recipient cell. Furthermore, RNA cargo in exosomes and microvesicles could hold tremendous potential as non-invasive biomarkers for multiple disorders, including pathologies of the immune system. This mini-review is aimed to provide the state-of-the-art in the EVs-associated RNA transcriptome field, as well as the comprehensive analysis of previous studies characterizing RNA content within EVs released by various cells using next-generation sequencing. Finally, we highlight the technical challenges associated with obtaining pure EVs and deep sequencing of the EV-associated RNAs.

## Introduction

The “Extracellular Vesicles (EVs)” is a general term used to describe various types of spheroid structures, encircled by a lipid membrane bilayer, which are secreted by mammalian cells either passively or upon certain stimuli (1). Since their initial discovery more than 30 years ago (2, 3) EVs have been purified from nearly all mammalian cell types including cells of the immune system (1). Furthermore, EVs have been detected in almost all human biological fluids, and shown to mediate cell-cell communication, thus playing a key role in the regulation of various physiological processes in the body (4) including the immune response (5–8). Finally, it becomes increasingly evident that EVs may contribute to carcinogenesis, as well as the spread of viruses, toxic proteins, and prions (1, 9).

**Figure 1:**
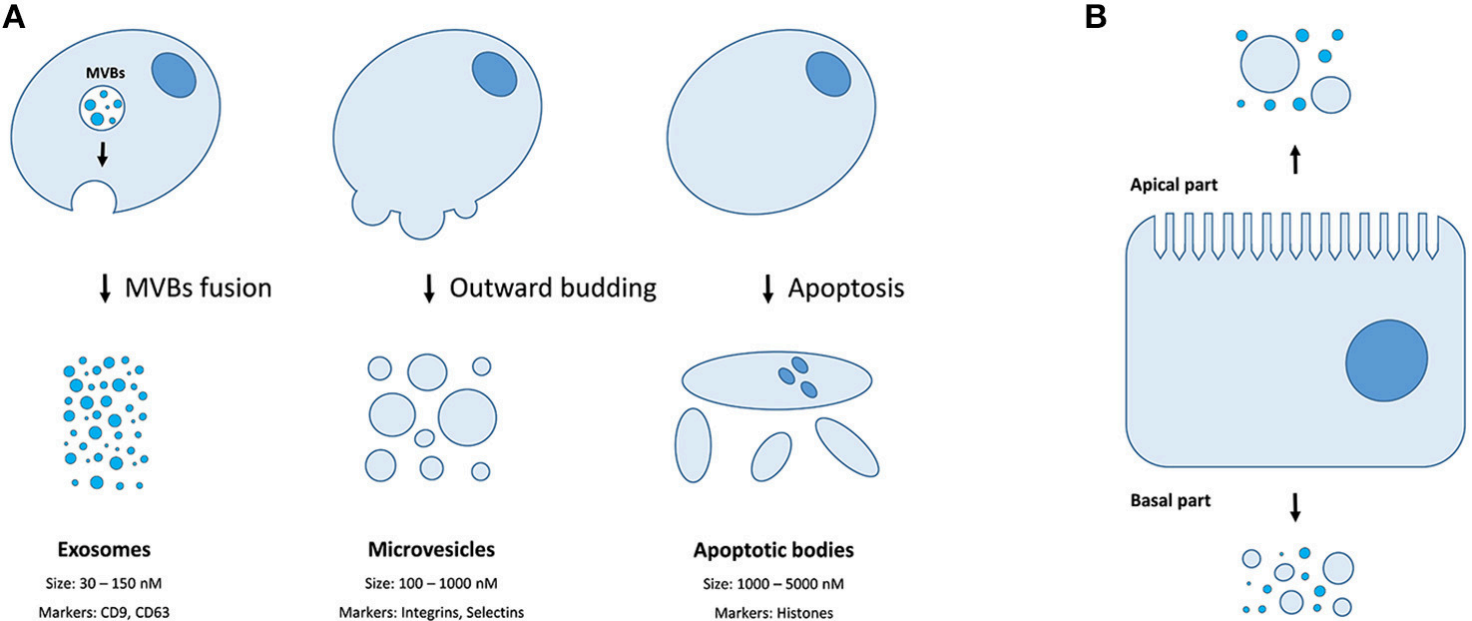
Extracellular membrane vesicles. **(A)** The mechanisms of generation, size distribution, and common protein markers of different EVs types; **(B)** Different populations of EVs could be released depending on the side of the plasma membrane of a polar cell.

There are three distinct types of EVs (as classified by their origin and biogenesis)—apoptotic bodies (ABs), microvesicles (MVs, also known as shedding vesicles), and exosomes (Figure 1A). The ABs are on average 1–5 μm in diameter and are by-products of cell disassembling during the apoptosis (10, 11). The MVs are formed by outward budding of the plasma membrane and are between 100 and 1,000 nm in diameter (8). The exosomes are the smallest type of EVs, having a diameter of 30–150 nm, and are primarily formed as intraluminal vesicles (ILVs) within multi-vesicular bodies (MVBs). Upon fusing of MVBs with the plasma membrane, the ILVs are released as exosomes into the extracellular space (8). Both MVs and exosomes contain various cytoplasmic and membrane-associated proteins as well as lipids, sugars, and nucleic acids (9), while ABs may in addition include nuclear fractions and cell organelles (1, 10, 11). Well-characterized protein markers for exosomes include various tetraspanins such as CD9, CD63, and CD81; while the MVs contain transmembrane proteins common for the plasmalemma such as integrins and selectins (8). Concurrently, ABs could be differentiated by the presence of histones (10, 11). Recently, large oncosomes (LOs) have been identified as the fourth type of EVs which are generated by shedding of membrane blebs from tumor cells and have a size similar to ABs (12).

Importantly, multiple research reports have demonstrated that various RNA species (including mRNA, miRNAs, and lncRNAs) entrapped within EVs can be transferred from donor to acceptor cells and interfere in gene expression in the latter (13, 14). This mini-review is aimed to provide a state-of-the-art in the EVs field, focusing primarily on the reported RNA cargo in different subtypes of EVs as well as the methodological challenges associated with purification of membrane vesicles and deep sequencing of their RNA content. Furthermore, we elaborate on a putative contribution of vesicular RNA to the functioning of the immune cells.

## The Challenges in Purification of EVs and Characterization of Extracellular RNA

The techniques widely used so far for isolating EVs include ultracentrifugation, density gradient flotation, ultrafiltration, chromatography, polymer-based precipitation, and immunoprecipitation (15). Differential ultracentrifugation is the most commonly used approach for EVs purification and, in particular, for separating exosomes from ABs and MVs (16). A biological fluid is first depleted from living cells, cell debris, ABs, and MVs with a series of lower-speed centrifugation, and the exosomes fractions are ultimately pelleted by ultracentrifugation. However, the final exosomes pellets can be contaminated with low-sized MVs, large protein aggregates as well as viruses (17, 18). The method of density gradient flotation harnesses differences in size, shape, and density of different EVs types and allows much higher purity of isolated EVs especially when combined with ultracentrifugation. However, high-density lipoproteins (HDL) and low-sized MVs are ultimately co-isolated with exosomes when using density gradient ultracentrifugation (19). Size exclusion chromatography generally allows recovery of EVs populations free from ribonucleoproteins and other soluble contaminants, however, different EVs types with a similar size could co-elute (20). While, ultrafiltration can also efficiently remove soluble components from EVs preparations, the similarly sized particles (both membrane vesicles and protein aggregates) will co-purify (21). An alternative approach that is increasingly being applied is the use of co-precipitants such as polyethylene glycol combined with low-speed centrifugation to aggregate and pellet exosomes for subsequent processing (22). However, while precipitation techniques have generally very high exosomes recovery rates, they also co-precipitate various proteins (23). Finally, immunoprecipitation techniques utilize antibodies against certain proteins located on the surface of EVs and can specifically isolate CD63, CD9, and CD81 positive exosomes (24). However, large-scale separation of exosomes with immunoprecipitation is challenging due to their highly diluted state in the biological fluids.

While each of the above-mentioned approaches harnesses certain differences in biophysical or molecular properties of EVs (including the size, the density, and the content of the surface proteins), neither method can recover a pure material and allows only an enrichment for certain subpopulations of EVs in a sample (15, 25). As a result, the characterization of EV type-specific RNA cargo remains highly challenging and strongly depends on the purification method. In addition, the bovine serum that is used as a component of most cell culture media could be a source of contaminating extracellular RNAs in a sample that mask human-derived RNA species having a sequence similar to bovine RNAs (e.g., miR-122) (26). The accuracy of the subsequent analysis of EV-associated transcriptome is also highly dependent on an RNA qualification method, including a DNA library preparation for deep sequencing (15). For instance, widely used commercial kits for RNA sequencing, by default, capture only 5′-phosphorylated 3′-OH short RNA molecules representing only a fraction of total RNA in the sample (27). Likewise, most whole-transcriptome sequencing techniques can incorporate only relatively long RNAs and, thus, overlook small RNAs.

Finally, certain cell types (e.g., cells of retinal pigmented epithelium and the intestine) exhibit a membrane polarity (Figure 1B). Therefore, EVs secreted by such cells might have distinct properties and molecular content depending on whether they derive from basal or apical parts of the membranes (28). While MVs and exosomes of polar cells have not yet been properly studied, the differences in structure, size, and lipid composition of apical and basal membranes could determine the features of the secreted EVs (28). The polarized trafficking machinery in certain cells suggests that additional care should be taken for isolating apical exosomes, including a careful control of the functional integrity of cell monolayers during preparation of conditioned media (29). On the contrary, an apical-only isolation approach risks missing important basolaterally released vesicles (28).

## The Reported RNA Contents Within Different EVs Classes

The presence of mRNAs, miRNAs, and lncRNAs within exosomes and MVs have been consistently shown with microarrays and RT-qPCR techniques in multiple early (13, 30–36) as well as more recent reports (37–40). The application of more advanced high-throughput RNA sequencing methods revealed the presence of various other RNA species within subpopulations of EVs isolated from biological fluids and cell conditioned media (Table 1). Those RNA species include snRNA, snoRNA, piRNA, vault RNA, Y-RNA, scRNA, SRP-RNA, and 7SK-RNA; as well as short fragments originating from rRNA, tRNA, mRNA, lncRNAs, and various intergenic repeats (40–57).

**Table 1:** The reports demonstrating a transcriptome content of the EVs using deep sequencing.

	**EVs source cells or biofluid**	**EVs isolation method**	**Expected EVs types**	**NGS type and platform**	**RNA classes and alignment statistics**
(41) PMID: 22821563	Co-cultures of dendritic and T-cells	DUC (S10 –> P100)	EXOs & small MVs	Small RNA-seq (Illumina)	Exons (6.7%) incl. small ncRNA (0.49%), introns (19.4%), genomic repeats (27.4%); Exons reads: protein coding (~84%), vault RNA (~3%), lincRNA (~2%), pseudogenes (~2%), SRP- RNA(~1%), rRNA (<1%), Y-RNA (<0.5%), miRNA (<0.5%), snRNA (<0.5%), snoRNA (<0.5%).
(42) PMID: 22965126	GT1-7 cells	DUC (S10 + 0.2 μm filtration –> P100)	EXOs & small MVs	Small RNA-seq (SOLID)	Genomic repeats (~50%), mRNA & ncRNA (~33%), small ncRNA (~15%), rRNA (~0.5%); Small ncRNA: tRNA (~90%), scRNA (~3%), siRNA (~2%), snRNA (~1%), miRNA (~1%), snoRNA (0.1%)
(43) PMID: 22849433	HEK293T cells	DUC (S2 –> P100)	EXOs, MVs & ABs	Small RNA-seq (Illumina) for miRNA only	Various miRNAs
(44) PMID: 23663360	Human plasma	Precipitation (ExoQuick)	EXOs & MVs	Small RNA-seq (Illumina)	miRNA (76.20%), rRNA (9.16%), DNA (5.63%), lncRNA (3.36%), mRNA (2.11%), piRNA (1.31%), tRNA (1.24%), snRNA (0.18%), snoRNA (0.01%)
(45) PMID: 23302638	Human saliva	UF (100 kDa) + GF	EXOs & small MVs	Small RNA-seq (Illumina)	miRNA (51-58%), rest: piRNA, snoRNA, genomic repeats
(46) PMID: 24255815	MDA-MB-231, MDA-MB-436 cells	DUC (S17 –> P100)	EXOs & small MVs	Total RNA-seq (Ion Torrent)	rRNA (~97%), protein coding (~1%), ncRNA (~1%)
(47) PMID: 23807490	U251 cells	DC (S1.8 –> P18)	Large MVs & ABs	Small RNA-seq (SOLID)	miRNA (38.7%), genomic repeats (>20%), rest: tRNA, vault RNA, miscRNA, intergenic and intronic
(48) PMID: 24352158	Human urine	DUC (S17 –> P200)	EXOs & small MVs	Small RNA-seq (Ion Torrent)	miRNA (35%), protein coding (3%), snRNA (0.02%), snoRNA (0.04%), lncRNA (0.19%), other non- coding RNAs (61%)
(49) PMID: 24816817	Human urine	DUC (S17 –> P118)	EXOs & small MVs	Total RNA-seq (Illumina)	rRNA (87%), protein coding (4.6%), ncRNA and genomic repeats (6.1%), mtRNA (0.1%)
(50) PMID: 26129847	Human mesenchymal stem cells	DUC (S10 –> P70)	EXOs & MVs	Small RNA-seq (Illumina)	tRNAs (23-50%), genomic repeats (17-40%), miRNA (2-5%), snoRNA (<0.6%), rest: miscRNA, rRNA, protein coding, snRNA, pseudogenes, mtRNA
(51) PMID: 26027894	HMEC-1 cells	DUC (S10 –> P100) + SDG	EXOs & small MVs	Small RNA-seq (Illumina)	miRNA (~80%), Y-RNA (~14%), mRNA (~1.5%), mtRNA (~1%), lncRNAs (~0.8%), vault RNA (~0.2%), other ncRNA (<0.1%)
(52) PMID: 25940616	MCF-7, MCF-10A cells	DC (S2 –> P16) DUC (S16 + 0.2 μm filtration –> P100)	Large MVs & ABs EXOs & small MVs	Small RNA-seq (Illumina)	Enriched in rRNA, Y-RNA, vault RNA, tRNA halves, much less miRNAs (<1%)
(53) PMID: 26176991	MML-1 cells	DC (S0.3 –> P2) DC (S2 –> P16.5) DUC (S16.5 + 0.2 μm filtration –> P120)	ABs Large MVs & ABs EXOs & small MVs	Small RNA-seq (Ion Torrent)	ABs: 3.3-6.5% miRNAs, rest: snRNA, snoRNA, mtRNA, Y-RNA, vault RNA MVs: 2.4-3.8% miRNAs, rest: snRNA, snoRNA, mtRNA, Y-RNA, vault RNA EXOs: 5.6-8.1% miRNAs, rest: snRNA, snoRNA, mtRNA, Y-RNA, vault RNA
(40) PMID: 27791479	HMC-1, TF-1 cells	DUC (S16.5 –> P120) + SDG	EXOs & small MVs (in LD and HD fractions)	Total RNA-seq and smRNA-seq (Illumina)	Total RNA-seq HD: protein coding (~75%), non-coding RNA (~25%); Total RNA-seq LD: protein coding (~20%), non-coding RNA (~80%); Small RNA-seq HD: miRNA (~23%), rest: vault RNA, snoRNA, snRNA; Small RNA-seq LD: tRNA (~28%), miRNA (~10%), rest: mtRNA, Y-RNA, piRNA
(54) PMID: 28381156	MKN45, SGC7901, NCI-N87, AGS, GES-1 cells	DUC (S2 –> P110)	EXOs, MVs & ABs	Small RNA-seq (Illumina)	miRNA (22-38%), rRNA (0.6-21%), snRNA (0.6-13%), rest: Y-RNA, piRNA, snoRNA, tRNA
(55) PMID: 29137313	HuH7, Hep3B, HepG2, HuH6 cells	DUC (S16 –> P120)	EXOs & small MVs	Total RNA-seq & small RNA-seq (SOLID)	Total RNA-seq: rRNA (32-66%), genomic repeats (15-44%), transcriptome (11-25%); Small RNA-seq: rRNA (16-54%), genomic repeats (24-40%), transcriptome (24-51%)
(56) PMID: 27858503	U87 cells	DUC (S10 –> P100)	EXOs & small MVs	Exome RNA-seq (Illumina)	Various mRNAs
(57) PMID: 30018314	HEK293T, RD4, C2C12, Neuro2a, C17.2 cells	DUC (S1.5 + 0.2 μm filtration –> P110)	EXOs & small MVs	Small RNA-seq (Illumina)	rRNA (~60%), small ncRNA (~22%), rest: tRNAs, protein coding, Y-RNA, miscRNA; Small ncRNA: piRNA (~33%), miRNA (~25%)

*S, supernatant obtained after centrifugation at the corresponding g (in thousands); P, pellet obtained after centrifugation at the corresponding g (in thousands); DC, differential centrifugation; DUC, differential ultracentrifugation; SDG, sucrose density gradient ultracentrifugation; UF, ultrafiltration; GF, gel-filtration; EXOs, exosomes; MVs, microvesicles; ABs, apoptotic bodies*.

In a pioneering work, Nolte-'t Hoen et al. characterized small RNA content in EVs released by the immune cells in culture using deep sequencing. Interestingly, the majority of total RNA isolated from EVs consisted of small RNAs (<200 nt), with minor amounts of 18S and 28S rRNA. Those short RNA fragments were primarily mapped to protein-coding regions and genomic repeats including SINE, LINE, and LTR sequences (Table 1). On the contrary, the majority of sequences present in the cellular small RNA population represented miRNAs, while the proportion of miRNAs in the daughter EVs was dramatically lower. Besides protein coding mRNA and repeats, the EV fractions contained all types of structural RNAs (such as vault RNA, Y-RNA, snRNA, snoRNA, SRP-RNA, and tRNA) as well as fragments deriving from lncRNAs and pseudogenes. Furthermore, many of the small non-coding transcripts were enriched in EVs relative to cellular RNA, indicated that cells might destine specific RNAs for extracellular release (41). A significant underrepresentation of miRNA over other RNA species in the exosomes released by various cultured cells have been also confirmed by multiple other studies (40, 42, 46, 50, 52, 55, 57). These data go in accordance with the previous observation that most individual exosomes does not carry any biologically significant number of miRNA copies (58). Nevertheless, other RNA sequencing experiments indicated that a significant proportion of small RNA-seq reads still correspond to miRNA in exosomes released by some cell lines (51, 53, 54, 57). Interestingly, several independent groups have observed a significant enrichment (15–50% of total reads) of RNA fragments mapped to genomic repeats comprising retroviral sequences, LTR, SINE, and LINE sequences (41, 42, 47, 50, 55). It has to be mentioned that the authors did not specify whether the small RNA library preparation protocols used in the above studies included the modifications to allow capturing 5′-OH and/or 3′-phosphorylated RNAs. Therefore, it remains unclear whether they actually characterized the full spectrum of small RNA in the corresponding EVs.

The sequencing of total (both long and small) RNAs in the EVs was reported by Jenjaroenpun et al. (46) and Miranda et al. (49) in the EVs present in conditioned media from MDA-MB cells and the urine, respectively, and showed a significant proportion of rRNA reads (87–97%) that was similar to the rRNA content in the cytoplasm. Out of the remaining 3–13% reads, approximately half was mapped to protein-coding transcripts while another half aligned to non-coding RNAs and genomic repeats. In another report by Beradrocco et al. the authors used both total RNA and small RNA sequencing protocols separately to characterize a spectrum of long RNAs encapsulated within the EVs released by four different liver cancer cell lines (55). The largest proportion (32–66%) of total RNA reads were mapped to rRNA, while 15–44% corresponded to the genomic repeats, and only 11–25% of reads were mapped to protein-coding and non-coding RNA genes. The small RNA sequencing performed on the same EVs preparations revealed only a slightly different distribution of RNA classes: rRNA (16–54%), genomic repeats (24–40%), and transcriptome (24–51%) (55). In another whole-transcriptome RNA-seq study paralleled with small RNA sequencing, Lasser et al. demonstrated that human mast and erythroleukemic cell lines release two exosomes populations (as separated by flotation on a density gradient into HD and LD fractions) (40). A clear lack of correlation between both long and short RNA cargo in HD and LD fractions suggests that extracellular RNA in these two fractions are associated with distinct pathways. Thus, reads mapped to mRNA transcripts were more abundant percentage-wise in the HD as compared to LD exosomes (75 vs. 20%), while the distribution of non-coding RNA reads was opposite (25 vs. 80%). In short RNA libraries, the HD fractions were enriched in mature miRNA (23%), while the LD fractions were dominated by tRNA (28%), and mature miRNA (10%) (40).

Another study investigated RNA content in three separate EVs types released by melanoma cells in culture and identified some non-coding RNAs to be enriched in every EV samples (53). Thus, RNA profiles indicated the presence of prominent 18S and 28S rRNA peaks in ABs and MVs with relatively moderate levels of small RNA. By contrast, exosomes contained predominantly small RNA and much less rRNA as compared to both ABs and MVs (53). Interestingly, a similar number of different miRNAs have been identified in every EV type. Overall, a close relationship between miRNA profiles was found in ABs and MVs (*R* = 0.91), MVs and exosomes (*R* = 0.86), as well as MVs and parental cells (*R* = 0.86). While a less strong correlation was found between ABs and exosomes (*R* = 0.79) and exosomes and cells (*R* = 0.75). Despite the fact that EVs subsets were different only to a minor degree from the aspect of their miRNA cargo, a significant number of miRNAs were detected only in exosomes and were absent in both ABs and MVs, supporting the concept of specific RNA loading into exosomes. It has to be mentioned that other ncRNA species were not only significantly more abundant as compared to miRNA but also selectively enriched in different EVs subtypes released by melanoma cells, which adds another level of complexity to investigating extracellular vesicle RNA cargo and its function (53).

Only a few reports have so far investigated small RNA cargo in EVs isolated from human biological fluids with next generation sequencing (44, 45, 48). These studies indicated that exosomes isolated from human plasma, saliva, and urine contained a significant proportion of miRNA reads (35–76% of total). The rest RNA species in the EVs from the above mentioned biofluids included fragments of rRNA, lncRNAs, tRNA, mRNA, repeated regions as well as small noncoding RNA such as piRNA, snRNA, and snoRNA. It is important to mention that exosome isolations from biofluids may contain much higher amounts of large protein aggregates, including miRNA-loaded AGO complexes that are normally released upon cell death (59), as compared to “few-days” cell conditioned media. Therefore, it remains to be validated whether the miRNAs detected in human biofluids were indeed associated with the EVs. Interestingly, deep sequencing of total RNA purified from urea exosomes (49) revealed drastically different transcripts distribution than that observed by Cheng et al. (48). Specifically, a substantial proportion (~87%) of total RNA reads was mapped to rRNA and only about 8% of reads aligned to non-coding RNA and DNA repeats, while the remaining ~5% of reads corresponded to protein-coding RNA (49). Conversely, the mapping statistics and reads distribution reported by Miranda et al. were similar to those obtained upon total RNA sequencing of exosomes from cell conditioned media (46, 55).

To conclude, the collective evidence evolving from the above mentioned studies (Table 1) argue that EVs released by most cells indeed carry significant amounts of non-coding and protein-coding transcripts, as well as their parts, that should be considered when studying the effects of extracellular RNA on recipient cells. The differences in EVs RNA cargo content among the reported studies might be explained in part by: (1) cell type-specific RNA expression differences; (2) different EVs and RNA isolation methods; and (3) the use of different NGS library preparation protocols and sequencing platforms.

## The Role of the EVs RNA Content for the Immune System

While it was consistently shown that the exchange of exosomes and microvesicles among different immune cells contribute to both adaptive and innate immune response, the impact of the EVs RNA cargo onto immune cells function remains obscure (60). Mittelbrunn et al. demonstrated that exosomes originating from T cells are loaded with certain miRNAs (e.g., miR-335) can be internalized by the antigen-presenting cells APCs at the immune synapses and that subsequently led to the reduction of target mRNA SOX4 expression (61). Likewise, miRNA transfer from one dendritic cell (DC) to another via EVs led to alterations in recipient-cell gene expression (62), and regulatory T cells reduce Th1 (CD4+ IFNg+) inflammatory responses by EV transfer of miRNA (especially let-7d) to Th1 cells (63). The discovery of the variety of different RNA species in MVs and exosomes secreted by the immune cells as well (41), added another level of complexity to the theory of cell-cell communication via cell-free RNA. In particular, very marginal levels of EVs-encapsulated miRNAs as compared to other RNA species not only question the contribution of miRNA to cell-cell communication but also suggests that other RNAs might play much more determining biological role.

Thus, previous experiments revealed that extracellular miRNA can activate Toll-like receptor (TLR) 8 signaling, which induces cytokine secretion, presumably by mimicking viral RNA (64). The TLRs are a family of innate immune system receptors which recognize various molecular patterns of microbial pathogens and induce antimicrobial immune responses (65, 66). Specifically, both free-floating AGO protein bound miRNAs and miRNA encapsulated in EVs have been hypothesized to mediate communication between immune cells via binding to extracellular or intracellular Toll-like receptors (TLRs) (64, 67). Among the major effects of the nucleic acids-mediated activation of intracellular TLRs is the induction of certain cytokines essential for the innate immune response. While multiple other reports link activation of TLR pathways and exosomes, it remains unclear whether the observed effects were indeed mediated by the encapsulated RNAs. However, the finding that intracellular TLRs located within endolysosomal compartments can bind both double-stranded and single-stranded nucleic acids derived from viruses and bacteria (68) strongly suggest that various RNA classes incorporated within the EVs could also activate the corresponding TLRs. Due to the largely sequence-independent impact of nucleic acids on the TLRs, it is feasible that more abundant non-miRNA classes could significantly contribute to such activation. Overall, it remains feasible that combined interactions of vesicular RNAs and TLRs within and between diverse immune and non-immune cells could contribute to the regulation of the complex nexus of immune responses.

## Conclusion and Future Perspectives

Massive parallel sequencing has enabled characterization of the whole spectrum of nucleic acids in a given sample, and was consistently applied to demonstrate the presence of the complex RNA cargo within EVs populations released by various cells. Interestingly, the intravesicular miRNAs (which were well-documented previously using microarray and qPCR-based methods) represented only a very marginal proportion as compared to other RNA species including various small non-coding RNAs, lncRNAs, and mRNA fragments. A putative biological impact of the EVs-associated transcriptomes remains to be validated; however, multiple studies indicated that, at least, exosomal miRNA could mediate communication among various cell types including the immune cells. In addition, EVs-encapsulated miRNAs have been shown to serve as highly specific biomarkers for various pathological conditions and correlate with the presence of malignant tumors. Indeed, exosomes carrying a tumor-specific miRNA repertoire have been consistently detected in the venous blood of cancer patients and mouse models. The collective finding that non-miRNA species are in fact much more abundant in the isolated EVs populations, suggests that they could serve as even more promising non-invasive biomarkers for cancer and/or other disorders.

## Author Contributions

ATo and ATu conceived the study. ATu prepared the tables and figures and wrote the manuscript. ATo and OD participated in final manuscript design, and provided experts' opinion on the content and critical revision.

### Conflict of Interest Statement

ATu is affiliated with the company SciBerg e.Kfm. ATo is affiliated with the company SRC BioClinicum. The remaining author declares that the research was conducted in the absence of any commercial or financial relationships that could be construed as a potential conflict of interest.
